# Development of an LNA-Based qPCR Assay for Detecting *Toumeyella parvicornis* (Cockerell, 1897) (Hemiptera: Coccidae) from Insect and Honeydew DNA

**DOI:** 10.3390/insects16090982

**Published:** 2025-09-20

**Authors:** Domenico Rizzo, Alice Downes, Claudia Gabriela Zubieta, Michela Moriconi, Chiara Ranaldi, Bruno Palmigiano, Antonio Aronadio, Linda Bartolini, Edson Bolige, Antonio P. Garonna, Elia Russo

**Affiliations:** 1 Laboratory of Phytopathological Diagnostics and Molecular Biology, Plant Protection Service of Tuscany, Via Ciliegiole 99, 51100 Pistoia, Italy; domenico.rizzo@regione.toscana.it (D.R.); a.downes@studenti.unipi.it (A.D.); claudiagabriela.zubieta@regione.toscana.it (C.G.Z.); michmoriconi@gmail.com (M.M.); chiara.ranaldi16@gmail.com (C.R.); bruno.palmigiano@regione.toscana.it (B.P.); antonio.aronadio@regione.toscana.it (A.A.); linda.bartolini@regione.toscana.it (L.B.); edson.bolige@gmail.com (E.B.); 2 Department of Agriculture, Food and Environment, University of Pisa, Via del Borghetto, 80, 56124 Pisa, Italy; 3 Department of Agricultural Sciences, University of Naples ‘Federico II’, 80055 Portici, Italy

**Keywords:** invasive pest, *Pinus* sp., environmental DNA, early detection, sap-sucking pest, qRT-PCR, soft scale insect

## Abstract

The invasive sap-feeding pest *Toumeyella parvicornis* (pine tortoise scale) is rapidly spreading across Europe, threatening pine ecosystems, particularly in forest–urban areas of Italy. In this scenario, early detection and monitoring strategies are critical to prevent new outbreaks and mitigate impacts in infested regions. Current surveillance is challenged by the lack of rapid, sensitive tools for indirect detection of this cryptic, canopy-dwelling pest, despite advancements in molecular diagnostics and environmental DNA (eDNA). Here, we established a highly specific qPCR assay using LNA probe chemistry for detecting *T. parvicornis* DNA from both adult insects and their excreted honeydew. DNA was successfully isolated/quantified from all tested matrices. We recorded average Cq values of 20.9 for insect specimens and 30.3 for collected honeydew samples. Targeting the *COI* barcoding region, the assay demonstrated excellent specificity in both in silico and in vitro tests, showing no cross-reactivity to other pine-associated taxa. The limit of detection for DNA isolated from insect was 64 fg/µL. This is the first diagnostic protocol to use honeydew as a matrix for indirect detection of *T. parvicornis*. Optimized for routine application by Plant Health Services, this eDNA-based tool offers a valuable approach for future monitoring of sap-sucking hemipterans in multiple environments.

## Simple Summary

We present a fast and highly specific molecular tool for detecting the invasive pine tortoise scale, *Toumeyella parvicornis*, a growing threat to *Pinus* sp. across Europe. This method allows for the identification of the species not only from insect specimens but also from its honeydew secretions, a non-invasive matrix for pest surveillance. By combining real-time PCR with advanced LNA probe technology, the assay provides accurate detection of pine tortoise scale, which develops in the hard-to-reach canopy of host pine trees. This is the first diagnostic approach to exploit honeydew traces for monitoring this sap-sucking insect, offering a practical solution for early diagnosis and rapid response.

## 1. Introduction

With increasing rates of global trade and the effects of climate change, invasive insect species have emerged as one of the most pressing threats to both managed and natural ecosystems, frequently causing severe ecological disruptions and significant economic losses [1,2,3]. Their successful establishment in non-native regions often leads to the destabilization of local ecosystems and requires strict monitoring and management measures to limit their spread and impact [4,5,6].

The invasive pine tortoise scale (PTS), *Toumeyella parvicornis* (Cockerell, 1897) (Hemiptera: Coccidae), has become a serious pest of *Pinus* species [7,8]. This sap-sucking insect is native to the Nearctic and has extended its distribution to parts of Europe, with Italy currently experiencing the most severe outbreak [8,9]. PTS colonizes the apical shoots of host pines and causes damage through direct phloem feeding and the abundant excretion of honeydew, which promotes the growth of sooty mold [8,10]. This dual effect reduces photosynthetic efficiency and contributes to decline of host trees [7,11]. Although its impact in its native range is low [12], accidental introductions can be severe [9], as was observed in the Antilles in the 2000s, where the pest almost led to the extinction of the native Caribbean pine (*Pinus caribaea* var. *bahamensis*) in the Turks and Caicos Islands [11]. Since its first detection in southern Italy in 2014 on *P. pinea* (stone pine) near Naples [13], PTS has spread rapidly across several Italian regions, mainly affecting coastal pine forests and having far-reaching landscape and ecological impacts [8,14,15]. The invasive potential of the pest has triggered regulatory attention, and it is currently inserted in the European and Mediterranean Plant Protection Organization (EPPO) Alert List [16]. Official phytosanitary measures in Italy include containment protocols such as delimitation surveys and the destruction of infested plants [17]. However, early detection and tracking of *T. parvicornis* remains a challenge as its developmental stages are cryptic, located high in the canopy and not easily accessible without specialized equipment (e.g., aerial platforms or cherry pickers). These logistical limitations highlight the urgent need for rapid, sensitive, and species-specific monitoring tools to improve PTS surveillance and enable timely responses by the Plant Health Service.

In the current era of molecular diagnostics, accurate and reliable methods have been developed to support the early detection of invasive insect species, particularly in contexts where direct visual identification is limited by cryptic life stages or inaccessible habitats [18,19,20,21]. Among these, real-time quantitative PCR (qPCR) assays are well-established molecular approaches that enable rapid and accurate identification of target species by detecting insect DNA even from their environmental traces [22,23,24,25]. Furthermore, these tools are non-destructive and adaptable to a wide range of environmental matrices [26,27,28,29]. Nevertheless, a validated molecular protocol for the detection of *T. parvicornis* is currently lacking, especially one that is suitable for both insect-derived and indirect environmental samples such as honeydew.

Here, we developed a real-time qPCR assay incorporating Locked Nucleic Acid (LNA) technology into the probe design for the specific and sensitive detection of *T. parvicornis* DNA. LNA-based probes technology was selected for its enhanced ability to discriminate between closely related nucleotide sequences and for its robustness in heterogeneous biological samples [30]. The assay targets the conserved region of the mitochondrial *cytochrome c oxidase I* (*COI*) gene and was optimized for application to both direct insect samples and honeydew residues. Performance evaluation was conducted according to EPPO standard PM7/98(5) [31], focusing on key diagnostic parameters including analytical specificity (inclusivity and exclusivity), sensitivity, repeatability, and reproducibility. The resulting protocol provides a reliable molecular tool for routine phytosanitary surveillance of PTS and paves the way for innovative diagnostic approaches targeting invasive sap-sucking pests.

## 2. Materials and Methods

### 2.1. Sample Collection from PTS

Adult specimens of *T. parvicornis* were collected from infested *P. pinea* twigs in outbreak regions of Tuscany and Campania, Italy. In Tuscany, twelve 10–15 cm-long infested twigs were sampled in the province of Pisa (43°43′08.59″ N; 10°16′54.42″ E) each colonized by approximatively five adult females at varying developmental stages. Samples were stored in sterile 50 mL tubes and preserved at or below –20 °C until used for diagnostic validation. PTS honeydew was collected from infested *P. pinea* in Castel Volturno (Campania region, 40°56′15.0″ N; 14°01′11.0″ E) using a modified protocol adapted from established methodologies [32,33]. Sampling occurred biweekly from late May to early July 2024, coinciding with peak insect activity [8]. For each infested tree, 5–6 terminal shoots (20–30 cm) were enclosed in sterile plastic bags and transported to the laboratory. Shoots were placed in plastic containers with 5 cm Petri dishes to collect honeydew excretions. Collected droplets were inspected for purity under a SteREO Discovery V8 stereomicroscope (Zeiss, Oberkochen, Germany) and then transferred into a sterile 2 mL centrifuge tube. An additional fraction was obtained by carefully scraping honeydew residues from needles using a sterile scalpel and the collected drops were centrifuged until they settled to the bottom of the tube. All samples were sealed with parafilm, stored at –80 °C, and later used for DNA extraction.

Honeydew-coated fallen needles were also collected from infested sites in Tuscany in June–July 2024. Aliquots (1–1.5 g) were prepared by cutting the needles into 2–3 cm fragments and stored for downstream molecular analysis.

### 2.2. Non-Target Samples for Specificity Testing

To assess analytical specificity, DNA was extracted from non-target insect species phylogenetically related to *T. parvicornis* or sharing similar ecological niches (Table S1). These were obtained from the entomological biomolecular collection of the Phytopathological and Molecular Biology Laboratory (Plant Health Service, Tuscany Region, Pistoia, Italy). Samples were preserved in 70% ethanol at room temperature.

### 2.3. DNA Isolation and Quantification

DNA was extracted using two different protocols depending on the sample type. Adult insects, including both *T. parvicornis* and non-target species, were processed with a 2% CTAB (cetyltrimethylammonium bromide) protocol using the Maxwell^®^ 16 Instrument (Promega, Milan, Italy) following the methodology described in [34]. Honeydew samples and honeydew-coated pine needles were instead processed using QuickExtract Plant DNA Extraction Solution (Lucigen, Middleton, WI, USA). For environmental matrices, approximately 1 cm fragments of needles or 20 µL of honeydew were combined with 1.4 mm zirconia beads in 2 mL microcentrifuge tubes and homogenized in a Retsch vibromill at 20 Hz for 10 s. Subsequently, 80 µL of extraction buffer was added, and the tubes were incubated at 65 °C for 10 min, followed by 95 °C for 10 min with intermittent shaking at 350 rpm. DNA extracts were further diluted 1:10 to 1:20 in cases where high debris content or turbidity was observed.

DNA extracted from adult insects was quantified using a QIAxpert spectrophotometer (Qiagen, Hilden, Germany). However, spectrophotometric quantification of honeydew-derived DNA proved unreliable due to interference from the extraction buffer components. Therefore, due to these analytical limitations and the need for a reliable indicator of DNA amplifiability from this complex biological matrix, the amplification efficiency of extracted DNA from honeydew samples was instead assessed using a probe-based qPCR targeting a conserved region of the eukaryotic 18S ribosomal RNA gene [35,36]. This approach does not provide absolute DNA quantification but rather serves as a functional proxy for DNA amplifiability and acts as a biological performance control. The assay was performed on a CFX96 thermal cycler (Bio-Rad, Hercules, CA, USA) following [24], with thermal cycling conditions and primer/probe sequences detailed in Table S2.

### 2.4. Primer and LNA Probe Design

**Figure 1:**
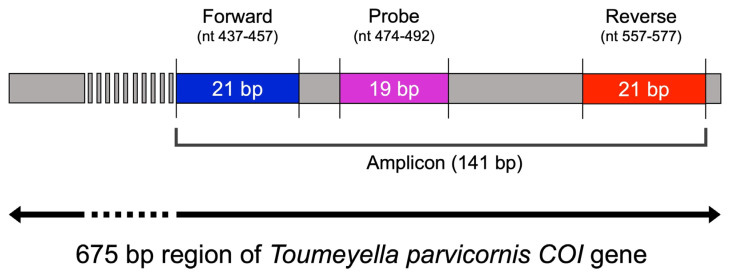
Schematic representation of the primer and LNA probe design targeting *Toumeyella parvicornis COI* gene. The forward primer (blue), reverse primer (red), and LNA probe (purple) were designed to amplify a 141 bp-specific fragment within this barcoding region (GenBank Accession: OR797509.1).

Primer pairs and LNA probes were designed using OligoArchitect™ software (Sigma-Aldrich, St. Louis, MO, USA), targeting a conserved region of the mitochondrial *COI* gene (GenBank accession: OR797509.1) specific to *T. parvicornis* (Figure 1, Table 1). Design criteria included melting temperature, GC content, absence of self-dimers (ΔG) and secondary structures, and amplicon size.

**Figure 2:**
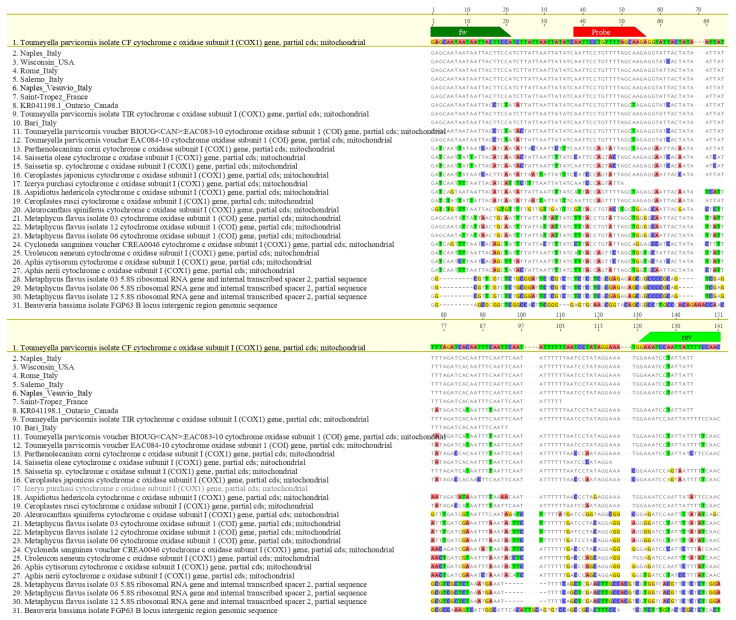
Alignments between the *Toumeyella parvicornis* amplicon and different sequences of other species phylogenetically close or occupying similar ecological niches or potentially morphologically confusable with *T. parvicornis*. Primers are indicated in green and LNA probe in red.

**Figure 3:**
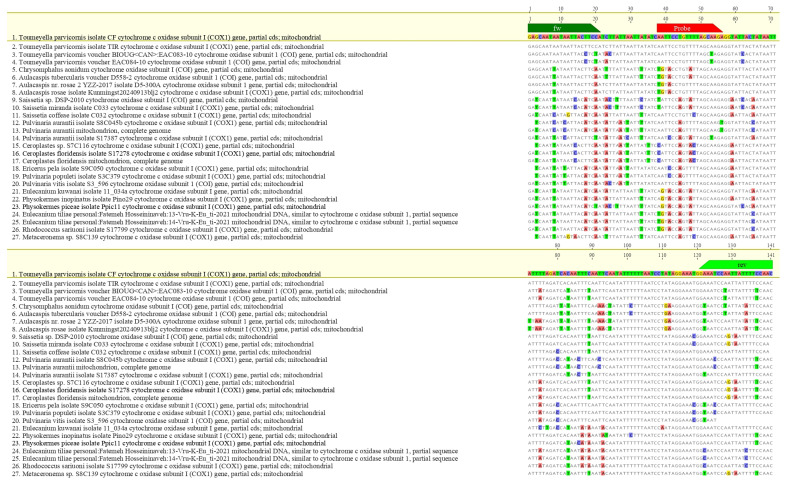
Alignments between the amplicon of *T. parvicornis* and the sequence of main organisms with the highest genetic plausibility from Blast analysis^®^ (Basic Local Alignment Search Tool) software. The primers are indicated in green and the LNA probe in red.

Oligonucleotides were synthesized by Eurofins Genomics (Ebersberg, Germany). In silico specificity was assessed via multiple sequence alignments using MAFFT v7 [37] integrated into Geneious 10.2.6 (Biomatters Ltd., Auckland, New Zealand). The parameters used were: scoring matrix 200 PAM/k = 2, gap open penalty 1.53 and offset value 0.123. The hypothetical amplicon based on the designed primers was subjected to further verification in BLASTn analysis (NCBI BLAST v2.14, accessed 18 April 2025). Inclusivity was evaluated by aligning global mitochondrial *T. parvicornis* sequences; exclusivity was tested against related taxa to verify absence of cross-reactivity (Figure 2 and Figure 3).

### 2.5. qPCR Assay Optimization

Optimization of the qPCR assay focused on maximizing sensitivity and specificity in complex matrices. Primer concentrations (0.2, 0.3, 0.4 µM) and LNA probe concentrations (0.1, 0.2, 0.3 µM) were tested using a thermal gradient (50–60 °C) on 5 ng/µL *T. parvicornis* DNA. Amplification was performed in a CFX96 real-time PCR system in 20 μL reactions using 0.2 mL PCR plates (Starlab, Milan, Italy). Each reaction included 2 μL of template DNA. Negative (no-template water), positive, and non-target controls were included in all runs. Ambiguous results were reanalyzed. Data were processed using CFX Maestro v2.3 (Bio-Rad, USA).

### 2.6. Assay Validation and Performance Characteristics

Validation of the molecular assay followed the EPPO PM7/98(5) standard, assessing analytical specificity, sensitivity, repeatability, and reproducibility (see Section 1). Specificity was confirmed through both inclusivity tests on geographically diverse *T. parvicornis* populations and exclusivity tests using DNA from non-target species (Table S1). Each sample was tested in triplicate, and in the case of inconclusive results, repeat testing was conducted until consistent outcomes were obtained.

Sensitivity was evaluated using DNA extracts from five adult *T. parvicornis* specimens normalized to 5 ng/μL. A series of 1:5 serial dilutions was prepared, ranging from 5 ng/μL to 2.56 fg/μL. Each dilution point was analyzed in triplicate, and a standard calibration curve was generated from the resulting Cq values. This curve was subsequently used to quantify *T. parvicornis* DNA in environmental samples, including honeydew collected in situ and laboratory-produced honeydew.

Repeatability was assessed using DNA extracts of adult *T. parvicornis* at 0.008 ng/μL in eight technical replicates, analyzed in triplicate within a single experimental run. Reproducibility was evaluated under the same conditions but across different days and by different operators. The variability in Cq values was measured and expressed as mean values with standard deviations (SD) [38,39].

## 3. Results

### 3.1. DNA Extraction from Different Samples

DNA was successfully extracted from both honeydew-contaminated *P. pinea* needles and laboratory-produced honeydew samples using the two extraction protocols described. Specifically, eight field samples of honeydew-smeared needles and twelve laboratory honeydew samples from *T. parvicornis* were processed. Each sample was extracted in triplicate, and the resulting DNA quality and concentration, based on absorbance ratios, were summarized in Table 2. Quantification was only possible for DNA extracts obtained via the 2% CTAB protocol using the Maxwell 16 automated extractor. In contrast, extracts produced with the QuickExtract Plant DNA Extraction Solution (Lucigen) yielded inconsistent absorbance readings. This discrepancy was likely due to chemical interference from the extract buffer components, which affected the A260/280 absorbance ratios, rendering direct spectrophotometric quantification unreliable. As a result, for these latter samples, the presence of amplifiable DNA and the efficiency of the extraction and amplification process was assessed indirectly through qPCR performance. Specifically, Cq values from q PCR amplification of the endogenous 18S ribosomal gene and the *T. parvicornis*-specific target gene were used to evaluate the suitability of the genomic DNA for amplification. Average Cq values for all tested matrices, including those reflecting DNA amplifiability, are reported in Table 2.

### 3.2. qPCR Assay Optimization with LNA Probe

**Figure 4:**
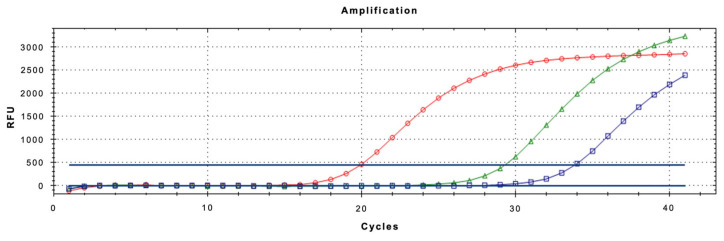
Amplification curves of *Toumeyella parvicornis* adults (spheres), honeydew produced in the laboratory (triangles) and needles with honeydew (squares).

The real-time qPCR assay using LNA probe was optimized to ensure maximum sensitivity and specificity, especially when analyzing complex matrices like honeydew. Optimization focused on primer/probe concentrations and thermal cycling conditions. All reactions were performed in a 20 µL volume containing 10 µL of QuantiNova™ 2x Supermix (Qiagen), 2 µL of template DNA, and varying concentrations of primers and probe. The best-performing configuration was found to be 0.4 µM for each primer and 0.2 µM for the LNA probe. The final thermal profile for the assay consisted of an initial denaturation at 95 °C for 2 min, followed by 40 amplification cycles of denaturation at 95 °C for 15 s and annealing/extension at 52 °C for 40 s (Figure 4). These conditions provided robust amplification across all tested matrices.

### 3.3. Assay Validation

**Figure 5:**
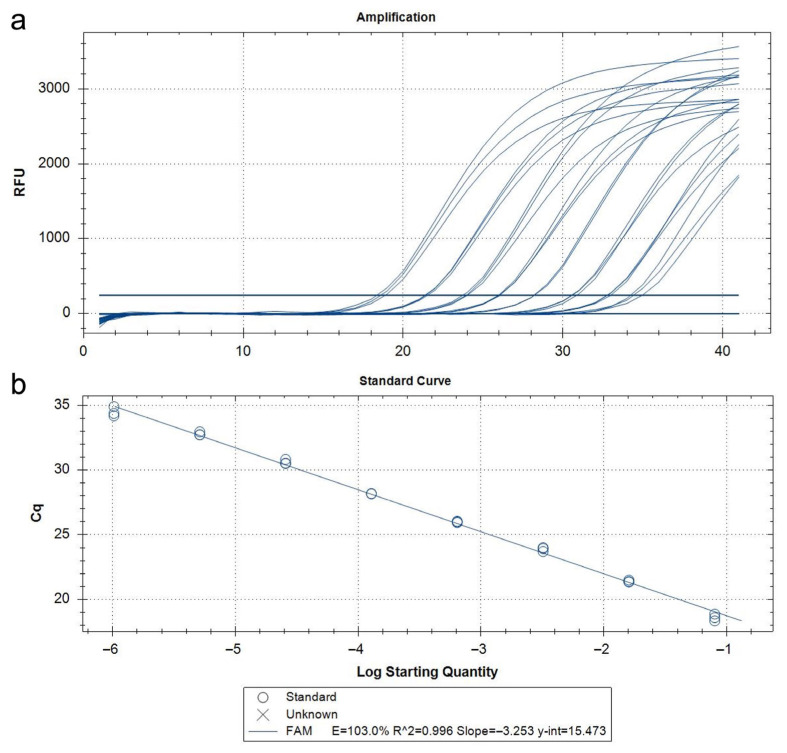
Amplification curves (**a**) and standard curves (**b**) relating to the qPCR probe assay using serial dilutions (1:5) of *Toumeyella parvicornis* DNA of insect adult from 5 ng/µL to 12.8 fg/µL.

The qPCR assay developed in this study demonstrated 100% analytical specificity, both in vitro and in silico. DNA extracted from target and non-target insect species (as listed in Table S1) produced no false positives or non-specific amplification. The results aligned with in silico sequence alignments that confirmed inclusivity across geographically diverse *T. parvicornis* populations and exclusivity against phylogenetically related taxa. No abnormal amplification curves or background noise were observed, and there was no need to apply Cq cut-offs to avoid misinterpretation. Analytical sensitivity, or the limit of detection (LoD), was determined using serial dilutions of *T. parvicornis* DNA extracts starting at a normalized concentration of 5 ng/μL. The standard curve derived from these dilutions demonstrated high amplification efficiency (103%), with an R^2^ value of 0.999, indicating excellent linearity (Figure 5, Table 3).

Relative quantification of *T. parvicornis* DNA in lab-produced honeydew and field-collected honeydew-smeared needles was achieved by comparing Cq values from these samples to the standard curve derived from the adult insect DNA dilutions. Quantitative results are presented in Table 4.

The assay also demonstrated excellent repeatability and reproducibility, both achieving 100% agreement. Repeatability was assessed using eight replicates of *T. parvicornis* adult DNA extracts, each tested in triplicate within a single run. Reproducibility was confirmed across independent runs performed on different days by different operators. In both cases, mean Cq values and corresponding standard deviations remained consistently low, as shown in Table 5 and Table 6, confirming the robustness of the method.

## 4. Discussion

Early detection and monitoring tools are crucial for limiting the introduction and spread of invasive species, providing an opportunity to prevent the negative impacts associated with their establishment by enabling rapid detection and supporting effective management responses [27,40,41]. The invasive sap-sucking pest *T. parvicornis* has recently emerged as a serious threat to *Pinus* sp. across some European countries, particularly in anthropized areas of the Italian peninsula [8,42]. As with many sternorrhynchan species, the small size and cryptic nature of PTS life stages, make logistically ineffective the traditional surveillance methods based on visual inspection and morphological identification. Additionally, the towering size of large part of the susceptible host pines further complicates access to canopy for routine diagnostics, especially in mature trees growing in natural or urban green spaces [43].

In this work, we have successfully developed and validated a real-time qPCR assay using LNA probe technology for the direct and indirect diagnosis of *T. parvicornis* from DNA of both insect and honeydew-contaminated samples, respectively.

Honeydew, a sugar-based matrix produced by sap-feeding pests, offers several advantages as a substrate for pest detection [44,45]. This matrix, which is secreted in large quantities during periods of high feeding activity, accumulates on surfaces under infested foliage and can be sampled in a non-invasive manner [46]. Recent field studies have demonstrated that the honeydew of the invasive planthopper *Lycorma delicatula* (Hemiptera: Fulgoridae) contains sufficient DNA to allow detection even at low densities, establishing a new frontier in monitoring of hemipteran pest [20,47].

While TaqMan-based qPCR has shown adaptability across diverse environmental matrices [18,25,41], including insect frass and other environmental residues [24,45,46,48,49], our decision to use LNA chemistry was driven by its superior diagnostic performance [50]. LNA probes incorporate modified ribose rings that “lock” the sugar backbone into a rigid conformation, making them particularly well-suited for genetic testing challenged by samples with low-quality or inhibitor-rich DNA [30,51].

The test developed demonstrated remarkable analytical performance. Specificity reached 100%, with no evidence of non-specificity or abnormal amplification curves. Specificity was confirmed both in vitro on a series of target and non-target species (characterized by genetic affinity or morphological similarity) and in silico using available sequences of *T. parvicornis* from around the world. The limit of detection (LoD) recorded was up to 64 fg/μL, comparable to the recent LNA-based test validated on DNA derived from adults of the quarantine pest *Agrilus anxius* (Coleoptera; Buprestidae) [30]. Furthermore, repeatability and reproducibility were confirmed by consistent Cq values with minimal variations in terms of standard deviation (SD) (less than 0.5) [52] between technical and biological replicates and independent tests. The developed protocol also proved effective across diverse environmental sources, including undiluted honeydew and honeydew-coated needles, confirming its robustness in complex backgrounds often challenged by PCR inhibitors. While genomic DNA was successfully isolated from all tested samples, reliable quantification via spectrophotometry was only achieved for CTAB-extracted specimens. Given the limitations of UV spectrophotometry for honeydew-derived DNA (likely due to extraction kit components), 18S-based qPCR was used as a proxy for assessing the suitability of extracted genetic material. Although this method does not directly quantify DNA concentration or purity, it provided a reliable indirect assessment of template amplifiability. Similar approaches for evaluating DNA quality and ensuring a positive signal from low-input samples have been adopted in biomolecular diagnostic assays for pest surveillance [30] and plant pathogen diagnosis [35,36].

A noteworthy limitation of our method could lie in the relatively higher synthesis cost of LNA probe compared to conventional TaqMan ones [30]. While this may limit uptake in low-budget surveillance programs, the long-term diagnostic reliability, specificity, and minimized need for additional testing (i.e., sequencing) justify the initial cost of investment. These efforts can be applied in recently invaded areas [53] to limit the further spread of the pest and prevent the huge infestation rates observed especially in southern Italy, where PTS has caused extensive ecological and landscape damage, particularly in Rome, killing tens of thousands iconic stone pine tree (*P. pinea*) [54].

By demonstrating that *T. parvicornis* can be accurately detected through its honeydew alone, this study represents a paradigm shift in diagnostic entomology. It supports the growing trend toward passive and indirect sampling strategies in the environmental DNA (eDNA) surveillance [44,55,56] and could inspire similar approaches for other sap-feeding pests such as mealybugs, aphids, and psyllids. Integrating these methods into phytosanitary monitoring frameworks could significantly improve the early detection rates of invasives [57], especially for canopy-dwelling or visually cryptic species [18,47].

## 5. Conclusions

This study provides a molecular protocol for detecting *T. parvicornis* using its honeydew as a diagnostic substrate. By targeting this biologically relevant and accessible matrix, the assay aligns with recent advances in terrestrial eDNA and paves the way for the development of non-invasive monitoring tools for sap-sucking pests. The high specificity and sensitivity achieved across complex environmental substrates underscore the assay’s suitability for integration into regional surveillance programs by Plant Health Services, particularly where direct insect sampling is logistically challenging. Future works should focus on combining this laboratory-based approach with eDNA collection strategies in field scenarios to enhance its applicability for early detection of invasive hemipteran pests.

## Figures and Tables

**Table 1:** Technical data (OligoArchitect Online) of *Toumeyella parvicornis* qPCR primers and LNA probe tested in this study.

**Sense Primer: GAGCAATAATAATTACYTCYA**				**Position: 437**
**Length (bp)**	**Tm (°C)**	**GC%**	**Self-Dimer (ΔG)**	
21	57.0	28.6	−2.7	
**Anti-Sense Primer: GTTGGAAAATAATWGGATTTC**				**Position: 577**
**Length (bp)**	**Tm (°C)**	**GC%**	**Self-Dimer (ΔG)**	
21	57.2	28.6	−1.8	
**Product:**				
**Rating**	**Length (bp)**	**Tm (°C)**	**Ta Opt (°C)**	**Cross Dimer (ΔG)**
73.5	141	69.7	52.0	−4.1
**Sense Dual-Labeled Probe: AAT(T)CC(T)GT(T)TT(A)GCWAGA**				**Position: 577**
**Length (bp)**	**Tm (°C)**	**GC%**	**Self-Dimer (ΔG)**	
19	64.2	31.6	−1.3	

**Table 2:** DNA extraction performance from different *T. parvicornis* samples. Results are based on the mean DNA concentration (±SD), absorbance ratio (A260/280) where measurable, and mean Cq values obtained using 18S qPCR and the LNA probe qPCR assay developed in this study.

Sample	Mean DNA Concentration (ng/µL)	Absorbance (A260/280)	Mean Cq (18S)	Mean Cq (qPCR LNA Probe)
Adults	123 ± 12.45	1.87 ± 0.04	20.2 ± 0.12	20.9 ± 0.19
honeydew-contaminated needles	-	-	24.2 ± 0.34	31.2 ± 0.87
laboratory-collected honeydew	-	-	25.4 ± 0.22	30.3 ± 1.25

**Table 3:** Serial dilutions (in triplicate—A, B, C) with detection limit (LoD) from adult DNA extract of *Toumeyella parvicornis*.

Dilutions	ng/µL	A	B	C	Cq Average	SD
-	5	18.76	19.11	18.9	18.92	0.18
−1	1	21.04	21.65	21.38	21.36	0.31
−2	0.2	23.67	23.93	23.79	23.80	0.13
−3	0.04	26.15	26.56	26.65	26.45	0.27
−4	0.008	28.44	28.92	29.57	28.98	0.57
−5	0.0016	31.06	31.83	32.4	31.76	0.67
−6	0.00032	34.17	35.03	34.56	34.59	0.43
−7	0.000064	36.56	37.03	38.82	37.47	1.19
−8	0.0000128	n/a	n/a	n/a	n/a	-

**Table 4:** 1:5 limiting dilutions, corresponding average Cq values and estimated amounts (in pg) of extracted DNA of *T. parvicornis* in different matrices investigated. Data were taken at the limiting dilutions and for a final volume of 20 µL.

Matrix	Dilution Limit	Cq Average	Estimated Quantity /20 µL
honeydew-contaminated needles	Third dilution 1:5	34.68 ± 1.67	36.3 ± 0.45 pg/µL
laboratory-collected honeydew	Third dilution 1:5	34.54 ± 0.97	23.3 ± 0.06 pg/µL

**Table 5:** Repeatability values indicating replicates and corresponding mean Cq ± SD values at a concentration of 0.008 ng/µL on extracted adult insect DNA of *Toumeyella parvicornis*.

Replicas	A	B	C	Cq Average	SD
1	28.95	29.5	29.02	29.16	0.30
2	28.85	29.55	29.12	29.17	0.19
3	28.78	29.13	29.05	28.99	0.18
4	28.8	29.42	29.18	29.13	0.31
5	28.79	29.07	29.63	29.16	0.43
6	28.82	29.42	29.2	29.15	0.30
7	28.84	29.68	29.07	29.20	0.43
8	29.04	29.56	29.18	29.26	0.27

**Table 6:** Reproducibility values indicating replicates and corresponding mean Cq ± SD values at a concentration of 0.008 ng/µL on extracted adult insect DNA of *Toumeyella parvicornis*.

Replicas	A	B	C	Cq Average	SD
1	29.27	29.68	29.28	29.41	0.23
2	29.43	29.62	29.57	29.54	0.10
3	29.39	29.62	29.32	29.44	0.16
4	29.19	29.48	29.42	29.36	0.15
5	29.24	29.39	29.56	29.40	0.16
6	29.12	29.89	29.43	29.48	0.39
7	29.2	29.41	29.04	29.22	0.19
8	29.34	29.58	29.22	29.38	0.18

## Data Availability

The original contributions presented in this study are included in the article/supplementary material. Further inquiries can be directed to the corresponding authors.

## Supplementary Materials

The following supporting information can be downloaded at: https://www.mdpi.com/article/10.3390/insects16090982/s1, Table S1. List of samples used in this study. Table S2. Primer/probe sequences, reaction mix, and thermal cycling conditions for the qPCR assay targeting the eukaryotic 18S rRNA gene, used to indirectly assess DNA amplifiability from samples prepared using the QuickExtract Plant DNA Extraction Solution.

## Abbreviations

The following abbreviations are used in this manuscript:

PTS	Pine tortoise scale (*Toumeyella parvicornis*)
EPPO	European and Mediterranean Plant Protection Organization
COI	Cytochrome c oxidase subunit I (mitochondrial gene)
18S	18S ribosomal RNA gene
CTAB	Cetyltrimethylammonium bromide
qPCR	Quantitative polymerase chain reaction
fg	Femtogram
pg	Picogram
LNA	Locked nucleic acid
LoD	Limit of detection
