# Supplementary material for: Development of an LNA-Based qPCR Assay for Detecting Toumeyella parvicornis (Cockerell, 1897) (Hemiptera: Coccidae) from Insect and Honeydew DNA
Source: Insects. 2025 Sep 20;16(9):982. doi: 10.3390/insects16090982 (PMC12470656; doi:10.3390/insects16090982)
Supplement: Supplementary file 1 [file insects-16-00982-s001.zip › insects-3749837-supplementary.pdf]

Table S1. List of samples used in this study.

Code ID	Organism	Supplier	Type	Matrix
MR 002281/1	*T. parvicornis*	PPS-T	Adult	Infested *P. pinea* twigs
MR 002281/2	*T. parvicornis*	PPS-T	Adult	Infested *P. pinea* twigs
MR 002281/3	*T. parvicornis*	PPS-T	Adult	Infested *P. pinea* twigs
MR 002281/4	*T. parvicornis*	PPS-T	Adult	Infested *P. pinea* twigs
MR 002281/5	*T. parvicornis*	PPS-T	Adult	Infested *P. pinea* twigs
MR 002281/6	*T. parvicornis*	PPS-T	Adult	Infested *P. pinea* twigs
MR 002281/7	*T. parvicornis*	PPS-T	Adult	Infested *P. pinea* twigs
MR 002281/8	*T. parvicornis*	PPS-T	Adult	Infested *P. pinea* twigs
MR 002281/9	*T. parvicornis*	PPS-T	Adult	Infested *P. pinea* twigs
MR 002281/10	*T. parvicornis*	PPS-T	Honeydew-smeared needles	Infested *P. pinea* twigs
MR 002281/11	*T. parvicornis*	PPS-T	Honeydew-smeared needles	Infested *P. pinea* twigs
MR 002281/12	*T. parvicornis*	PPS-T	Honeydew-smeared needles	Infested *P. pinea* twigs
MR 002281/13	*T. parvicornis*	PPS-T	Honeydew-smeared needles	Infested *P. pinea* twigs
MR 002281/14	*T. parvicornis*	PPS-T	Honeydew-smeared needles	Infested *P. pinea* twigs
MR 002281/15	*T. parvicornis*	PPS-T	Honeydew-smeared needles	Infested *P. pinea* twigs
MR 002281/16	*T. parvicornis*	PPS-T	Honeydew-smeared needles	Infested *P. pinea* twigs
MR 002281/17	*T. parvicornis*	PPS-T	Honeydew-smeared needles	Infested *P. pinea* twigs
MR 002281/18	*T. parvicornis*	UniNa	Honeydew	Lab-produced samples
MR 002281/19	*T. parvicornis*	UniNa	Honeydew	Lab-produced samples
MR 002281/20	*T. parvicornis*	UniNa	Honeydew	Lab-produced samples
MR 002281/21	*T. parvicornis*	UniNa	Honeydew	Lab-produced samples
MR 002281/22	*T. parvicornis*	UniNa	Honeydew	Lab-produced samples
MR 002281/23	*T. parvicornis*	UniNa	Honeydew	Lab-produced samples
MR 002281/24	*T. parvicornis*	UniNa	Honeydew	Lab-produced samples
MR 002281/25	*T. parvicornis*	UniNa	Honeydew	Lab-produced samples
MR 002281/26	*T. parvicornis*	UniNa	Honeydew	Lab-produced samples
MR 002281/27	*T. parvicornis*	UniNa	Honeydew	Lab-produced samples
MR 002281/28	*T. parvicornis*	UniNa	Honeydew	Lab-produced samples
MR 002281/29	*T. parvicornis*	UniNa	Honeydew	Lab-produced samples
MR 000801	*Aleurocanthus camelliae*	PPS-T	DNA extract	Adult
MR 001683	*Aleurocanthus camelliae*	PPS-T	DNA extract	Adult
MR 001684	*Aleurocanthus camelliae*	PPS-T	DNA extract	Adult
MR 001916	*Aleurocanthus camelliae*	EuGen	gStrand	Gene sequence
MR 000239	*Aleurocanthus spiniferus**	PPS-T	DNA extract	Adult
MR 001697	*Aleurocanthus spiniferus**	PPS-T	DNA extract	Adult
MR 001747	*Aleurocanthus spiniferus*	UniNa	DNA extract	Adult
MR 001799	*Aleurocanthus spiniferus*	CREA-DC	DNA extract	Adult
MR 002045	*Aleurocanthus spiniferus*	EuGen	gStrand	Gene sequence
MR 000807	*Dialeurodes citri**	PPS-T	DNA extract	Adult
MR 001685	*Planococcus citri**	UniPi	DNA extract	Adult
MR 001686	*Planococcus citri*	UniPi	DNA extract	Adult
MR 001687	*Planococcus citri*	PPS-T	DNA extract	Adult
MR 000847	*Planococcus ficus**	UniPi	DNA extract	Adult
MR 000241	*Ricania speculum**	PPS-T	DNA extract	Adult
MR 000286	*Saissetia oleae**	UniFi	DNA extract	Adult
MR 000290	*Saissetia oleae*	PPS-T	DNA extract	Adult

*Identified by sequence analysis of *COI* gene, using primers LCO1490/HCO2198 (Folmer et al., 1994). UniFi = University of Florence, Italy; UniPi = University of Pisa, Italy; UniNa = University of Naples, Italy; PPS-T= Plant Protection Service of Tuscany, Italy; CREA_DC = Consiglio per la Ricerca in agricoltura e la analisi dell'Economia Agraria, Italy; EuGen = Eurofins Genomics.

Table S2. Primer/probe sequences, reaction mix, and thermal cycling conditions for the qPCR assay targeting the eukaryotic 18S rRNA gene, used to indirectly assess DNA amplifiability from samples prepared using the QuickExtract Plant DNA Extraction Solution.

Reaction Mix

Reagents	Final concentration	Mix for 1 sample
Sterile water for molecular biology	-	5,54 µL
QuantiNova Probe PCR Kit Master Mix (Qiagen)	1 X	10.0 µL
Primer forward 18S_Uni_F 20 µM	0.28 µM	0.28 µL
Primer forward 18S_Uni_R 20 µM	0.28 µM	0.28 µL
Probe 18S_Uni_P (FAM) (10 µM)	0.18 µM	0.36 µL
BSA 50 µg/µL	0, 4 M	0,1 µL
	Tot.	18 µL

qPCR primers

Name	Nucleotide sequence	Reference
18S UNI_F	GCAAGGCTGAAACTTAAAGGAA	Ioos et al., 2009
18S UNI_R	CCACCACCCATAGAATCAAGA	Ioos et al., 2009
18S Uni_P	FAM_ACGGAAGGGCACCACCAGGAGT_BHQ1	Ioos et al., 2009

Thermal cycle

	Temperature	Time x cycle	
Denaturation	95 °C	2’	1
Amplification	95 °C	10”	40
	58 °C	40”	
